# Effectiveness and acceptability of a novel school-based healthy eating program among primary school children in urban Sri Lanka

**DOI:** 10.1186/s12889-021-12041-8

**Published:** 2021-11-13

**Authors:** Sumudu Nimali Seneviratne, Sanathanee Sachchithananthan, Pavithra Sewwandi Angulugaha Gamage, Renuka Peiris, Vithanage Pujitha Wickramasinghe, Noel Somasundaram

**Affiliations:** 1 Department of Paediatrics, Faculty of Medicine, University of Colombo, Colombo, Sri Lanka; 2 Sri Lanka Diabetes and Cardiovascular Disease Initiative, Colombo, Sri Lanka; 3 Ministry of Education, Battaramulla, Sri Lanka; 4 Ministry of Health, Nutrition and Indigenous Medicine, Colombo, Sri Lanka

**Keywords:** Obesity, Overweight, Schoolchildren, Food diary, South-Asia, Healthy-eating

## Abstract

**Background:**

Obesity rates are rising rapidly in low-middle-income-countries (LMICs). School-based interventions have shown moderate efficacy in improving diet and lifestyle associated with obesity in high-income countries. However, there is little data available on effective interventions suitable for LMICs. We devised a novel program for primary school children including a simple storybook and sticker-based food-diary (FD) and conducted a pilot study to evaluate the acceptability and short-term effectiveness of the program.

**Methods:**

This pre-post intervention study included grade 1 and 2 students from four public schools in Colombo, Sri Lanka. Weight and height were assessed, and participating children self-monitored their diet using sticker-based FDs for one week at baseline (pre-test). The following week, class teachers discussed the storybook, which incorporated the benefits/disadvantages of a healthy/unhealthy diet and lifestyle in classrooms. At the end of the intervention, participating children were self-monitoring their diet again for a week (post-test). A simple scoring system was used to derive a weekly score based on the healthiness of the meals consumed each week (FD-score). The primary outcome of the study was change in eating habits following the story book discussion (post-test FD score - pre-test FD score). Acceptability and effectiveness were also assessed by anonymized feedback questionnaires for parents and teachers.

**Results:**

One thousand and forty-two students completed the program. There was an improvement in eating habits of participating children, with FD scores improving by 12% from 51 ± 23 at baseline to 63 ± 24 following the intervention (*p* < 0.001). Further, when considering BMI category of participants: 69.1% were normal weight (NW), 18.3% underweight (UW), 7.4% overweight (OW) and 5.2% obese (OB). Improvement in eating habits were seen among children of all BMI categories (change in FD-score: UW 13.2%, NW 12.3%, OW 10.4% and OB 12.4% (*p* < 0.001)).

Furthermore,> 90% parents(*n* = 1028) and > 95% teachers(*n* = 39) strongly agreed/agreed that the intervention was easy to implement, motivated children and led to an observable improvement in healthy eating.

**Conclusion:**

This novel program led to an immediate improvement in eating habits and was well accepted by parents and teachers making it a potentially suitable intervention for wider implementation in primary schools in urban Sri Lanka.

**Supplementary Information:**

The online version contains supplementary material available at 10.1186/s12889-021-12041-8.

## Introduction

Childhood overweight (OW) and obesity (OB) have increased rapidly over the past few decades, initially among high income countries and subsequently among low-middle-income-countries (LMICs) [1, 2]. Global prevalence of OW/OB in children/adolescents aged 5–19 years increased from 4 to 18% between the four decades from 1975 to 2016, and it continues to rise in LMICs, especially in urbanised areas [3]. Mass production of highly processed ready-to-eat foods and mass advertising strategies targeting children and adolescence by the global food industry have contributed to this increase [4, 5]. The World Health Organization advocates developing and disseminating appropriate nutrition information for children in a simple, understandable and accessible manner to effectively counteract these adverse factors and combat childhood obesity [6]. Thus, there is a need for innovative interventions to educate children about healthy and unhealthy foods in an interesting way, to encourage and re-instill healthy eating habits among children and adolescents. A community based approach where messages on healthy lifestyle change is delivered to large numbers of children would be most appropriate and cost-effective [7].

Schools offer a unique setting to implement large scale interventions to improve healthy nutritional practices and healthy body weight [2]. According to the World Health Organization (WHO), school-health interventions are an efficient way to impart health education and bring about behavioural changes to improve healthy lifestyle of young people [8]. It has been established that OB/OW can be successfully prevented through school-based programs during an early age and lead to long-term health benefit in preventing obesity related non-communicable diseases (NCD) [9–11].

Given the recent rapid rise in obesity and related NCDs in LMICs, there is a growing and urgent need for effective and feasible school-based obesity prevention programs which are appropriate and feasible for LMICs [12]. There are, however, no consensus or guidelines on the most effective methods to improve dietary habits in school children. Further, most studies on effective school based interventions have been reported from high income countries, and may not be feasible or appropriate for LMICs [10, 13]. When considering previous studies on school-based interventions for primary school children reported from LMICs many have small study samples and have not been feasible for larger scale implementation [9, 12, 13].

Prevalence of obesity and type 2 diabetes is rising rapidly in Sri Lanka, especially among young age groups [14–17]. A recent survey showed prevalence of OW and OB of 14.7% among 14–15-year-old school children in Colombo, Sri Lanka, while a community-based study conducted in 2019, among 5–18-year-olds from the Western Province showed OW and OB rates of 11.3% and 10.3% respectively, with 11.2% also showing impaired fasting glucose and 4.3% having impaired glucose tolerance [14, 17]. Unhealthy food habits including increased consumption of fast food and soft drinks, reduced consumption of vegetables and fruits, frequent visits to restaurants and watching television while eating have been reported as risk factors associated with OW/OB in Sri Lankan school children [18]. In a qualitative study, Sri Lankan school principals from government schools reported: lack of knowledge about the healthy food choices among students; a national curriculum with less focus on nutrition; and inadequate knowledge on nutrition in teachers since the curriculum doesn’t demand it; as major barriers to preventing obesity in school children [19]. In Sri Lanka, children are not provided with meals from schools. They either bring their meals from home or purchase it with pocket money. Although fast food is restricted in school canteens, children often tend to purchase and consume processed food outside/near school environment [19]. Further, while differences in food diversity, and variations in carbohydrate and protein consumption have been reported among different ethnicities in Sri Lanka [20, 21], move away from locally produced traditional foods is occurring, due to food advertisements and easy availability of highly processed and advertised foods in many parts of the country [19].

In Sri Lanka, free state education is provided for children from grade 1 to 13 in all national and non-national government schools. National schools are a sector of public schools which have more facilities and gaining admission to such schools is highly competitive, and they tend to include children from higher socioeconomic groups [22]. Higher rates overnutrition have been reported among children attending national schools compared to non-national schools in Sri Lanka [22]. The Ministries of Health and Education share a joint responsibility in implementing the school health programs in government schools for children and adolescents attending government schools. The School Health unit of the Family Health Bureau works with the ministries of education and health and national organizations to coordinate support to meet the healthy development needs of school children [23].

The Sri Lanka Diabetes and Cardiovascular Disease Initiative (SLDC), a 3-year national level project with multiple stakeholders including Ministry of Health, Ministry of Education, and Family Health Bureau was initiated in 2017 in Sri Lanka, to prevent and control diabetes and other NCDs by strengthening primary care services and health promotion. The SLDC school health team developed a novel intervention to educate and motivate young primary school children in grade 1 and 2, to be conscious and mindful about healthy eating and to empower them as agents of change, for a healthier lifestyle within their own families. We focused on national schools in Colombo for this pilot study, as the main aim of the program was obesity prevention and higher prevalence of obesity is reported from national schools in urban Sri Lanka in comparison to non-national schools where undernutrition is more prevalent [22]. In this manuscript, we describe details of this novel program, as well as baseline data and the immediate effects of this intervention on eating habits of young primary school children in four national schools in Colombo, Sri Lanka, and perceptions of teachers and parents of participating students on its feasibility, acceptability, and effectiveness.

## Methods

The study followed a pre-post study design, assessing the same group of students at baseline and immediately post intervention, with a plan for longer term follow-up. The study was conducted in four national state-schools in Colombo, Sri Lanka in 2018–2019. The schools were chosen by multi-stage random sampling among state national schools in Colombo district based on Ministry of Education school statistics 2017. The program was conducted as a co-curricular activity program for grade 1 and 2 students in the selected schools, in accordance with the Declaration of Helsinki, with the approval of the Ministry of Education (ED/01/21/02/06). Permission was obtained from school authorities, class teachers and parents after educating them about the program at a special school meeting held in each school, prior to implementation.

### Novel tools developed for the intervention

**Fig. 1:**
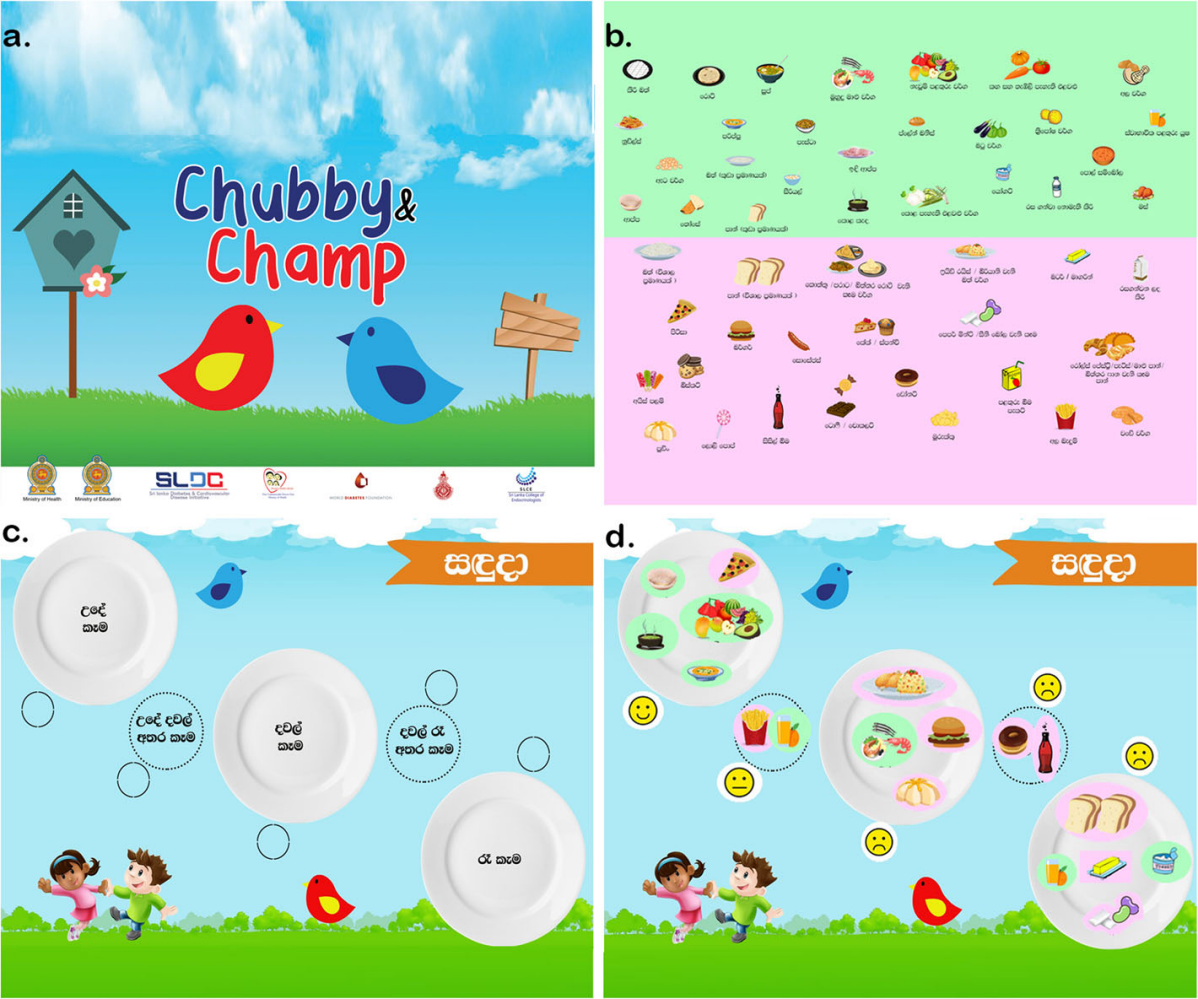
a. - Cover page of the story book; b. - Food sticker page; c.-Food intake page; d.-Completed food intake page

Two novel educational tools were developed for this program: A 12-page colourful picture story book (Fig. 1a.) and a food diary (FD) (Fig. 1 b.-d.). The storybook was developed to illustrate the adverse effects of an unhealthy lifestyle, and benefits of a healthy diet and lifestyle in a child-friendly age-appropriate manner suitable for children aged 5–7 years schooling in grade1 and 2 in Sri Lanka, by child health and educational professionals with expertise in school health, nutrition and childhood obesity from the Department of Paediatrics, Faculty of Medicine, University of Colombo, and the Ministry of Education, Sri Lanka. Care was taken to make the story interesting and motivational for participating children and encourage positive role modelling. Pictures were used to reinforce the messages and encourage discussion in the classroom. Language was used in a manner understandable and acceptable for a young audience. The objectives were to establish self-motivation to induce healthy behaviour change, and a sense of control over their own health.

The FD was similarly developed with guidance from a panel of experts in childhood nutrition, child health and primary education. It contained an instruction page, 14 daily food intake pages, 14 food sticker-pages (Fig. 1b.), a smiley-face sticker page (containing happy, neutral and sad faces), and a scoring page. The sticker pages contained more than 50 food items/groups commonly consumed by the community, with healthy food items placed against a green background and unhealthy food items against a red background (Fig. 1b.). Foods known to contain high sugar/fat content, and highly processed ready-to-eat foods were taken as unhealthy foods. The daily food intake pages consisted of a plate format consisting of 5 plates, depicting main meals and snacks (Fig. 1c.). Each child was instructed to record the food items they consumed daily by pasting the relevant stickers on the plate format for a week at baseline, and again, a week after the story discussion (example given in Fig. 1d.). When the sticker was pasted, the background colour remained as a rim around each food item (Fig. 1d.). Each meal/snack (plate) was graded with + 1 mark (happy face) if it contained more green foods; and − 1 marks (unhappy face) if it contained more red foods and 0 marks (neutral face) if it contained equal amount of red and green foods and for empty plates zero score was assigned. From this, a weekly FD score was derived by obtaining the sum of all the scores obtained throughout the week. As each week consisted of a total of 35 meals/snacks, (5 per day for 7 days), the weekly FD score was calculated and converted to a percentage score dividing the total score by 35. A more positive FD score would indicate a healthier diet. The aims of the FDs were to: 1) educate the child in an age-appropriate manner about healthy /unhealthy food items using a simple colour coding scheme 2) encourage mindful eating and raise consciousness on food consumed at each meal/snack; 3) engage parents/family and raise awareness on family food habits indirectly by helping the child maintain FD at home; and 4) provide the child with a sense of responsibility/ownership for adopting a healthier lifestyle/diet.

### Feedback questionnaires for parents and teachers

Two anonymised feedback questionnaires were prepared: one for participating parents and the other for class teachers. The aim of the questionnaires was to determine parents’ and class teachers’ perceptions on the feasibility, acceptability and effectiveness of the tools and the processors used.

The parents’ questionnaire consisted of 6 graded response questions using a 5-point Likert scale ranging from strongly agree to strongly disagree, followed by an optional open area for comments and suggestions. The first four questions dealt with feasibility and acceptability and the last two questions were on effectiveness (parental perception of the benefit of the program) (see Table 2). The teachers’ questionnaire contained six graded response questions using a 5-point Likert scale similar to parental questionnaire and an optional open area for comments and suggestions. First four questions were used to assess the feasibility and acceptability of the program whereas the last question was on perceived effectiveness of the program (see Table 3).

### Study population and sampling method

The study population included grade 1 and grade 2 students from national schools in the Colombo district between 2018 and 2019. Four schools were chosen by multi-stage random sampling based on gender and instruction medium based on Ministry of Education school statistics 2017. A majority of national schools in Colombo are single gender schools and focus on one main medium of instruction. As differences in food habits due to ethnicity and gender may also influence the efficacy of the intervention, one school each from each medium of instruction and gender were selected to ensure appropriate ethnic and gender representation in this study.

### The intervention

Initially, height and weight of each participating student was measured using a wall-mounted stadiometer to the nearest 0.1 cm without shoes, while the weight was measured with lightweight clothing and without shoes to the closest 0.1 kg with an electronic body fat monitor scale (SF1515FPS). Then, participants self-monitored their diet using the FD for one week (pre-test) at home. Food stickers were pasted by the children based on their daily dietary intake. The activity was designed to be attractive to children and the parents were educated at the beginning of the program to allow and encourage the children to complete the activity themselves.

Each class teacher was provided with a story book, and the teachers read and discussed the storybook, in their respective classrooms, during the following week. The week after, students maintained the FDs for another week (post-test). The completed FDs were returned to class teachers, together with feedback forms filled in by parent/s. The class teachers were provided with a mark sheet and requested to enter the FD scores of their class students using the students class registration number. The study investigators collected the mark sheets, FDs and feedback forms from the respective schools, and conducted the data analysis.

### Data analysis

Participants Body Mass Index (BMI) was calculated using the formula weight/height^2^ (kgm^− 2^). Age was obtained by using the date of birth from the school register, height-for-age (HAZ), weight-for-age (WAZ) and BMI-for-age (BAZ) z scores were calculated, using WHO Anthroplus v.1.0.4 software. Nutritional status (underweight < −2SD, normal weight -2SD to +1SD, over weight + 1SD to +2SD and obese > +2SD) were determined per WHO Growth Reference 2007 [24].

The primary outcome of the study was change in eating habits following the story book discussion (Post-test FD score - Pre-test FD score). Subgroup analysis was conducted based on nutritional status (normal weight/ underweight/ overweight/obese), gender, and grade. The significance of the change was estimated using paired sample T tests, and significance level set at 0.05. Feedback from parents and teachers were summarised by frequency and percentage.

## Results

### Demographic feature of study population

Thousand and forty-two students completed the program. Mean age of participants was 6.3 ± 0.6 years (range 5.1–7.5). There was appropriately equal representation from grade 1 and grade 2 students (grade 1 = 497 (47.7%) and grade 2 = 545 (52.3%). There were 641 male students (61.5%), and 401 female students (38.5%).

Mean BMI was 14.6 ± 2.2 (range: 8.7–28.5). Number of students in each BMI category was as follows: underweight – 162 (18.3%), normal weight – 613 (69.1%), overweight – 66 (7.4%), obese – 46 (5.2%).

### Change in food habits following the intervention (FD scores)

Mean pre-test FD score was 51.4 ± 23.1% and the mean post-test FD score was 63.0 ± 24.5%. There was a mean increase in FD score by 11.6% (95% CI 10.3–13.0%, P < 0.001) (Table 1). Furthermore, on subgroup analysis, significant improvement in FD scores was seen among children of both grades, both genders and all BMI categories (See Table 1).

**Table 1:** Pre and post-test food diary (FD) scores and change in FD scores for the total cohort, and according to grade, gender, and BMI category (mean change and 95% confidence intervals)

Category	Variable	N (% of total)	Pre-test FD Scores%		Post-test FD Scores%		Δ FD Scores% (Post -Pre)	95% CI	P
			Mean	SD	Mean	SD			
**All**		1042 (100%)	51.4%	23.1	63.0%	24.5	11.6%	10.3%,13.0%	0.000
**Grade**	Grade 1	497 (47.7%)	52.7%	22.7	62.9%	24.4	10.2%	8.2%,12.0%	0.000
	Grade 2	545 (52.3%)	50.1%	23.4	63.1%	24.5	13.0%	11.1%,14.9%	0.000
**Gender**	Male	641 (61.5%)	50.3%	24.1	63.3%	25.2	13.0%	11.2%,14.7%	0.000
	Female	401 (38.5%)	53.0%	21.2	62.5%	23.2	9.5%	7.3%,11.6%	0.000
BMI category*	UW	162 (15.6%)	50.5%	23.5	63.7%	25.8	13.2%	9.7%,16.7%	0.000
	NW	613 (58.8%)	51.2%	22.6	63.5%	23.7	12.3%	10.5%,14.0%	0.000
	OW	66 (6.3%)	50.2%	23.0	60.6%	22.6	10.4%	5.2%,15.7%	0.000
	OB	46 (4.4%)	47.7%	25.1	60.1%	25.1	12.4%	6.9%,17.9%	0.000

*Excluding 155 (14.9%) students whose BMI data was not available

### Feedback from parents

We received feedback from 1028 parents and Table 2 summarises the responses to the parent feedback questionnaire. As shown in Table 2, more than 90% of parents agreed/strongly agreed that the FD was easy for their child to understand and follow, while more than 80% of parents agreed/strongly agreed that the children understood and wanted to adopt a healthier diet after listening to the story. Further, more than 85% agreed/strongly agreed that it motivated and led the child and family to adopt a healthier diet. Table S1 summarises parents’ suggestions & comments. The most frequent observations by parents were that their child had obtained a good understanding of healthy and unhealthy food (*n* = 93), refused to eat unhealthy food (*n* = 203), and showed increased motivation to eat healthy food (*n* = 59) (see Table S1). Many also said the FD was child-friendly and practical, and the child could do it on his/her own (*n* = 63), and that the program motivated parents to prepare healthy family meals and created an impact on the whole family (*n* = 58). Suggestions for improvement included adding more information on calorie value of food items (*n* = 15) and requests for longer/ continued use of the FD as a means of reinforcing healthy lifestyle in their child (*n* = 37). The only difficulty/problem identified by some parents was difficulty in maintaining the FD due to busy lifestyle (*n* = 10).

**Table 2:** Analysis of parents’ responses to the parental feedback questionnaire assessing feasibility, acceptability and effectiveness of the program (*n* = 1028)

No	Questions	Strongly Agree/ Agree		Not Sure		Disagree/ Strongly Disagree	
		n	%	n	%	n	%
**1**	FD instructions were easy to follow	987	96.0%	13	1.3%	28	2.7%
**2**	Happy to maintain FD for 2 weeks	946	92.0%	61	6.0%	21	2.0%
**3**	Child remembered and understood the story	904	87.9%	113	11%	11	1.1%
**4**	Child wanted to adopt healthier diet after hearing the story	867	84.3%	107	10.4%	54	5.3%
**5**	Observed improvement in food habits in child	903	87.8%	105	10.2%	20	2.0%
**6**	Parents felt motivated towards adopting a healthier diet for the family	970	94.3%	50	4.9%	8	0.8%

### Feedback from teachers

Table 3 and Table S2 summarises responses obtained via feedback questionnaires from the 39 class teachers who participated in this program. All the participating teachers (100%) strongly agreed/agreed that the instructions were clear, and easy to follow. Further, more than 95% also strongly agreed/agreed that the storybook was a good and adequate medium to educate students. However, several teachers (15%) found it difficult to find time to mark the FDs. Nearly, 95% teachers strongly agreed/agreed that they observed an improvement in food habits at the end of the program. Teachers’ suggestions included showing the story as a cartoon or using flash cards and providing some form of appreciation (star chart) for children consuming a healthier diet (Table S2).

**Table 3:** Analysis of teachers’ responses to the teacher feedback questionnaire assessing feasibility, acceptability and effectiveness of the program (n = 39)

No	Questions	Strongly Agree/ Agree		Not Sure		Disagree/ Strongly Disagree	
		n	%	n	%	n	%
1	Instructions were easy to follow	39	100.0%	0	0.0%	0	0.0%
2	Able to find time to mark the FDs	33	84.6%	4	10.3%	2	5.1%
3	Story discussion was an effective method of providing health information to children	39	100.0%	0	0.0%	0	0.0%
4	Children were interested in the story	39	100.0%	0	0.0%	0	0.0%
5	Children actively participated in the story discussion	38	97.4%	1	2.6%	0	0.0%
6	Saw an improvement in food habits of children after the intervention	37	94.9%	2	5.1%	0	0.0%

## Discussion

The aim of this novel primary school intervention was to educate and motivate young school children to adopt healthier eating patterns and empower them as agents of change within their own families. The study results showed that the intervention was effective in causing an immediate improvement in eating habits among grade 1 and 2 students from participating schools. Further the program was effective in children of all BMI categories. Feedback from parents and teachers on acceptability, feasibility and effectiveness of the program was positive, with more than 80% parents agreeing that the program was easy to follow and resulted in the child and whole family adopting a healthier diet and lifestyle. Further, more than 80% of participating teachers agreed that the program was easy to follow, appropriate, useful, and interesting for their students and 95% agreed/strongly agreed that it led to an observable improvement in student’s food habits. These findings suggest that this novel program is an effective and acceptable way of improving eating habits of young school children aged between 5 to 7 years.

Programs with active learning and student-centred activities appear effective in motivating primary school children to adopt healthy behaviour change, while self-learning and self-monitoring reduces the burden on teachers and parents [25]. A few previous studies, which used a story book to educate and motivate young children for behaviour change, found that properly constructed stories can be effective methods of encouraging positive behaviour change in young children [26–28]. For example, a study done in England for children aged between 7 and 11 years, using student-centred learning activities including art, quizzes and songs, led to decreased consumption of carbonated drinks, and a moderate reduction in overweight/obesity among students [29]. Further, a study in Thailand which used a story-based intervention including five story books and five e-books effectively decreased inappropriate behaviour of preschool children with autism [26]. Our study findings too indicate that interactive learning using a story book discussion and a picture-based FD are effective methods of educating and motivating young school children. Based on both parental and teachers’ feedback, the children appeared to have been interested in, and successfully grasped the health messages provided, by reading and discussion of a story book, by the class teacher, as a classroom activity. Further, the program motivated and empowered them to make healthy dietary/lifestyle changes, as depicted both by the reported improvement in eating habits, and the positive feedback from parents and teachers.

Positive parental involvement has also been reported previously as a factor contributing to the success of school-based lifestyle interventions in children [30, 31]. Positive family engagement is likely to result in more successful lifestyle change rather than individual efforts [9]. In our study, we attempted to engage parents/family and raise their awareness on family food habits indirectly, by requesting parents to guide their child to maintain the FD at home for two weeks daily. Further, we also requested parents to support their child if he/she initiated/requested a change in family lifestyle/diet after the story book discussion. Therefore, we involved the parents/family in the program indirectly, while maintaining the child as the main participant and message bearer between the school and home. These factors are likely to have contributed to the positive effects observed in our study.

Further, our study findings also open up the possibility of using school-based programs to improve eating/lifestyle habits of the rest of the family members as well. Recently school-based cluster randomized controlled trial was conducted among grade 8 students of 20 schools in Colombo district Sri Lanka to enable school children to act as change agents on weight, physical activity and diet of their mothers. Trained health promotion facilitators discussed NCD risk factors such as obesity, sedentary behaviour and unhealthy diet, and encouraged students to discuss these issues in groups as applicable to their homes, and come up with ideas on how to influence them [32]. In the 12-month follow-up survey Sri Lankan mothers whose child was in the intervention group showed a statistically significant reduction in weight and increase in physical activity compared with the control group of mothers whose child did not receive any intervention [32]. This study reflects and reinforces the concept that children are capable agents of change for healthy lifestyle changes within their families and concurs with our study finding of more than 90% of parents agreeing/strongly agreeing that they were motivated towards adopting a healthier diet for the family following the study.

When comparing our study findings, a few previous school-based intervention studies for primary school children from other LMICs have shown promising results, while ours in the first school-based intervention study to be conducted among primary school children in Sri Lanka, to the best of our knowledge. Positive change towards a healthier was reported from the Indian study MARG, large school-based intervention program among 80,000 Indian children aged 8–18 years from 50 urban schools, where a six-month educational intervention about optimal dietary and lifestyle practices, administered using innovative and age-appropriate education strategies and informative booklets, by special teams consisting of physicians, nutritionists and field workers, led to a significant improvement of knowledge and behaviour among participants, with regards to health, nutrition and NCDs [33]. One of the main differences between our study and the MARG study was the fact that the Indian intervention was administered weekly by a special team outside teaching hours, whereas our program was implemented during school hours by class teachers. An intervention comprising of eight fortnightly meetings using games, puppets, posters, music and children’s stories to educate 2nd graders at a public school in Brazil, on food groups, use of the food pyramid to construct snack menus, and physical activity education etc., led to an improvement in students’ eating habits as assessed by a 3-day food registration questionnaire, completed by the student in the classroom, with the help of teachers, to capture food and drinks consumed during school hours [34]. Another study in Trinidad, using a short term intervention where children were educated on nutritional value of food and physical activity over one month, was also found to be effective [35]. In this program, lessons on nutrition and physical activity were delivered by specially trained teachers. Students’ diet was assessed by a modified food frequency questionnaire while physical activity was self-reported for one week. The results showed improvement in the children’s knowledge and awareness on issues related to nutrition and physical activity [35]. It is noteworthy, that the above-mentioned programs/interventions utilised specially trained teachers/ other professionals for implementation. Our program, however, was carried out by class-teachers without prior training, and thus could be in-cooperated more easily into the school curriculum, making it more cost effective and sustainable for wider and longer term implementation, especially for relatively low resource settings.

Limitations of this study include lack of a control group, lack of pre validated data collection tools and lack of uniformity in the intervention administered by different class teachers according to their different teaching style. This study was conducted mainly to assess the effectiveness of this program in real world circumstances and to determine its suitability for wider implementation as a health promoting project in schools in Sri Lanka. Heterogeneity in administration of the intervention would be an integral part of such a program and thus, we believe that lack of uniformity in the classroom intervention allowing for some independence for the teachers was important to better reflect the true ground situation. Having a control group would have enhanced the scientific validity of these findings. Further, one could argue that feedback from parents and teachers is subjective, and we have attempted to make it more objective by using Likert scales and making feedback anonymous. The results reported here should be considered in the light of these limitations. Further it should be noted that the results presented here are preliminary and report on immediate outcomes following the intervention. Changes in food habits observed in this initial follow up period may be short lived as it will take longer time to establish lasting change in food habits. Longer follow up studies are needed to establish if the intervention was successful in initiating lasting change in food habits in this cohort. However, the positive feedback received from parents and teachers as well as the significant increase in the FD scores observed argue well for longer term positive change. We anticipate following up the participants to assess the long-term impact of this program on dietary habits and BMI.

Regarding generalizability of our study findings, the students participating in the study appeared to be representative of grade 1 and 2 children attending public national schools in Sri Lanka, with underweight rates similar to that reported among grade 1 students in Colombo district (18.9%), and national level data (19.4%) [23], and higher rates of over-nutrition in comparison to rates reported among grade 1 students in Colombo district (OW - 4.1%) and countrywide (OW-2.1%; OB-1.2%), reflecting the higher rate of over nutrition reported among Sri-Lankan schoolchildren attending public national schools previously [22, 23]. In Sri Lanka there is equal gender representation among school children (Male - 49.7%, Female - 50.3%) [36]. The higher representation of male students in our study sample simply represents the higher number of total student population in the two schools with male students chosen for this study, while gender-based subgroup analysis showed positive results indicating effectiveness of the intervention among children of both genders.

## Conclusions and recommendations

In conclusion, this novel program appears to be an acceptable and feasible school-based intervention to encourage healthier eating habits and lifestyle in young primary school children and initial data on its effectiveness and acceptability appears promising. Follow up studies which are ongoing to establish the effects of the program on longer term eating habits and BMI will help determine longer term effectiveness. If the follow up studies also show sustained positive results in-cooperation of this program into the school curriculum, together with supportive school policies would be an important recommendation for maintaining sustained healthy lifestyle changes among children [2, 37]. There is precedence where management of OW and OB has been included in the school curriculum previously in response to the high prevalence of OW/OB among grade 7 and grade 10 students in Sri Lanka, where the Family Health Bureau has provided technical guidance to the ministry of education and the national institute of education in the curriculum preparation [23]. Therefore, there is a pathway by which the SLDC can work with the multiple stakeholders of the project to implement lasting changes. Ideally, programs such as these should be supported by policy changes restricting, distribution and promotion of unhealthy food to school children, especially in the vicinity of schools [38, 39].

## Supplementary Information

**Additional file 1: Table S1.** Additional suggestions and comments from parents and their frequency. **Table S2.** Additional suggestions and comments from teachers and their frequency.

## Data Availability

The datasets used and/or analysed during the current study are available from the corresponding author on reasonable request.

## Abbreviations

LMIC: Low-Middle-Income Country
FD: Food Diary
NW: Normal weight
UW: Under weight
OW: Overweight
OB: Obesity
WHO: World Health Organization
NCD: Non-communicable disease
SLDC: Sri Lanka Diabetes and Cardiovascular Disease Initiative
BMI: Body Mass Index
HAZ: Height-for-age
WAZ: Weight-for-age
BAZ: BMI-for-age
SD: Standard Deviation
