# Supplementary material for: Effectiveness and acceptability of a novel school-based healthy eating program among primary school children in urban Sri Lanka
Source: BMC Public Health. 2021 Nov 13;21:2083. doi: 10.1186/s12889-021-12041-8 (PMC8590231; doi:10.1186/s12889-021-12041-8)
Supplement: Supplementary file 1 — Additional file 1: Table S1. Additional suggestions and comments from parents and their frequency. Table S2. Additional suggestions and comments from teachers and their frequency. [file 12889_2021_12041_MOESM1_ESM.docx]

**Table S1** Additional suggestions and comments from parents and their frequency

| **Remarks** | **n** | **%** |
| --- | --- | --- |
| Child refused to eat unhealthy food in the red section | 128 | 12.4% |
| Child got a good understanding on healthy and unhealthy food | 93 | 9.1% |
| Child refused to eat unhealthy food /did not allow us to buy unhealthy food on outing/special occasions | 75 | 7.3% |
| Child showed increased motivation to eat healthy food (grains, green leaves, vegetables) | 59 | 5.7% |
| The FD is child friendly and practical for children | 35 | 3.4% |
| We (parents) were motivated to prepare healthy food | 32 | 3.1% |
| Child was able to do the FD on his/her own | 28 | 2.7% |
| Project has created an impact on the entire family | 26 | 2.5% |
| *Suggest continuing FD for more than 2 weeks* | 25 | 2.4% |
| No problems encountered in pasting smiley faces | 24 | 2.3% |
| *Suggest including information on calories in the FD* | 15 | 1.5% |
| *Suggest maintaining FD each term for reinforcement of learning* | 12 | 1.2% |
| Child has understood the message conveyed from the story | 12 | 1.2% |
| Child taught the importance of healthy food habits to other family members by retelling the story at home | 11 | 1.1% |
| Difficult to maintain the FD due to busy lifestyle | 10 | 1.0% |
| Excellent way to educate small children | 6 | 0.6% |

(Observations/comments are given as normal text, and suggestions are given in italics)

*n=number of parents who made this remark*

| **Remarks** | **n** | **%** |
| --- | --- | --- |
| Difficult to do the teacher’s evaluation because of the time limitation | 10 | 25.6% |
| Child understood the importance of having healthy food | 7 | 17.9% |
| Children found the story interesting | 7 | 17.9% |
| Children said they asked their parents to prepare healthy food | 6 | 15.4% |
| Children wanted to become like the healthy character in the story | 4 | 10.3% |
| *Suggest showing story as animated cartoon / using flash cards* | 4 | 10.3% |
| *Suggest appreciating children who eat healthy food using star charts* | 3 | 7.7% |
| Children stopped buying unhealthy food from canteen | 2 | 5.1% |
| Children refused unhealthy food (ice-cream) during the class trip | 1 | 2.6% |

**Table S2** Additional suggestions and comments from teachers and their frequency

(Observations/comments are given as normal text, and suggestions are given in italics)

*n=number of teachers who made this remark*
